# Rural–urban differences in the economic contributions of veterinary practices across 10 states

**DOI:** 10.3389/fvets.2026.1779555

**Published:** 2026-02-26

**Authors:** Clinton L. Neill, Jonathan Baros, Abby ShalekBriski

**Affiliations:** 1 Department of Population Medicine and Diagnostic Sciences, Cornell University College of Veterinary Medicine, Ithaca, NY, United States; 2 College of Agriculture and Life Sciences, Virginia Polytechnic Institute and State University, Blacksburg, VA, United States; 3 Department of Agricultural Economics, Oklahoma State University, Stillwater, OK, United States

**Keywords:** economic contribution, IMPLAN, labor, rural, veterinary economics

## Abstract

**Objective:**

Quantify and compare the economic contributions of veterinary services across rural and urban counties in 10 U.S. states, and assess whether rural status is associated with higher veterinary economic intensity.

**Methods:**

We conducted a county-level economic contribution analysis of veterinary services using 2023 IMPLAN data. For each county, we extracted direct employment and output and calculated total effects (direct + indirect + induced) and multipliers. Counties were classified by rural-urban status, and log-linear OLS models estimated employment and output per veterinary establishment per 1,000 residents, controlling for county poverty and unemployment rates and state effects.

**Results:**

Across the sample, veterinary services generated 75,438 direct jobs and 97,912.70 total jobs, and $7.20B in direct output and $11.94B in total output. Rural counties accounted for 8,010 direct jobs and $706.90M in direct output vs. 67,428 direct jobs and $6,489.58M in urban counties. Employment multipliers ranged from 1.25 to 1.38 and output multipliers from 1.57 to 1.70. In regression models, rural status was positively associated with higher employment intensity and output intensity.

**Conclusion:**

Veterinary services generate substantial local economic activity with meaningful spillovers. Although urban counties dominate in total scale, rural counties show higher standardized intensity, supporting continued policy initiatives that target rural workforce recruitment and retention.

## Introduction

The veterinary profession supports animal health and agricultural production as well as local economies. Beyond providing clinical services in communities, veterinary practices generate employment opportunities, labor income, tax revenues, and downstream spending in regional supply chains and households (1, 2). Through purchases of pharmaceutical, diagnostics, medical equipment, and other local goods, veterinary practices generate upstream demand and induce household spending, creating multiplier effects that amplify regional impact (3, 4). The magnitude and structure of these effects likely vary across space. Rural veterinary practices operate in thinner labor markets characterized by lower veterinarian density (5) and documented recruitment and retention challenges (6), while urban practices exhibit higher service density and more specialization (7). Taken together, these contrasts motivate testing whether veterinary practices generate different levels of economic intensity across rural and urban counties.

Veterinary practice performance is influenced by broad macroeconomic conditions, including household income and inflationary pressures that affect willingness to pay for care (8). Yet most existing evidence is aggregated, and we have limited understanding of how veterinary economic activity—and the spillovers it generates—varies across rural and urban communities. Recent peer-reviewed work reinforces that contemporary access constraints in veterinary services are fundamentally place-based, operating through both workforce dynamics and spatial barriers. A large cross-sectional survey of U.S. veterinarians found that intent to stay/leave practice was broadly similar across rural and nonrural settings, but rural veterinarians reported longer work weeks, more on-call time, and different community-amenity tradeoffs—factors that can affect effective local service capacity even without differential “exit” rates (9). County-level mapping studies further document systematic geographic disparities in access to veterinary care, offering empirical evidence that travel time, provider distribution, and local socioeconomic conditions jointly shape the likelihood of receiving care (10). In parallel, recent syntheses on veterinarian burnout emphasize that reduced professional effort and workforce strain can translate demand pressure into capacity constraints, reinforcing the importance of measuring how local veterinary capacity links to broader community outcomes (11). Together, this emerging literature strengthens the case for county-level, rural–urban evidence that goes beyond headcounts to quantify the broader local economic stakes of veterinary service availability.

This literature gap matters because economic analyses of veterinarian location, spatial density, and shortage designations consistently point to geographic maldistribution (12), such that veterinarians cluster in larger markets, while rural coverage depends on local demand conditions, amenities, and policy incentives. This pattern creates comparative shortages that can constrain service capacity in rural communities (13, 14).

These frictions are often most acute in rural areas, where veterinarian density is lower and recruitment and retention challenges persist (5, 6). As a result, some rural communities have experienced contraction in local veterinary capacity. Policy initiatives such as the Veterinary Medical Loan Repayment Program (VMLRP) have sought to address shortages in rural and food animal medicine for more than a decade (15). However, stronger, place-based evidence on what a rural veterinary practice contributes to its county economy—relative to an urban or suburban practice—can inform the design and justification of federal, state, and local efforts to support rural veterinary services.

Conceptually, rural–urban differences in economic contribution can arise through several mechanisms. Rural practices often serve larger geographic catchments with higher travel and transaction costs, and they may act as essential service anchors in thinner markets where alternative providers and suppliers are limited. This can change both the scale of activity captured locally and the structure of spillovers: spending that is retained within the county may generate larger indirect and induced effects, while leakage to outside suppliers can dampen multipliers. At the same time, urban practices operate in denser markets with greater specialization and supplier networks, which may raise total scale but not necessarily standardized intensity.

To address these issues, this study analyzes rural–urban differences in the scale, structure, and intensity of veterinary economic activity in the United States. Regional input–output (I–O) models such as IMPLAN provide a systematic framework for estimating direct, indirect, and induced economic effects of veterinary services (2, 3). I–O methods have been used to assess service-sector contributions and regional spillovers in the economic literature (4, 16). Building on this literature, we use county-level data to estimate the economic contribution of veterinary practices in terms of employment and total economic output and then quantify rural–urban differences through standardized intensity measures and econometric comparisons.

Accordingly, we address two research questions:

(RQ1) How do the direct and total (direct + indirect + induced) economic contributions of veterinary services differ between rural and urban counties in the study states?

(RQ2) Is rural status associated with higher veterinary economic intensity (measured as employment and output per veterinary establishment per 1,000 residents) after controlling for county socioeconomic conditions and state effects?

## Materials and methods

To quantify the economic contributions of veterinary services at the county level, we employ a regional input–output (I–O) modeling framework using IMPLAN 2023 data. I–O models trace the flow of goods and services among industries, households, and institutions, providing a systematic accounting of how expenditures in one sector propagate through the broader regional economy (1). The IMPLAN system operationalizes Leontief-style I–O analysis and is grounded in Bureau of Economic Analysis benchmark tables, regional purchase coefficients, and trade-flow adjustments (2).

We implement an economic contribution analysis, rather than an economic impact analysis, because the veterinary sector is an existing and geographically distributed industry embedded within all U.S. counties. Contribution analysis does not answer the counterfactual question of what economic activity would occur in the absence of veterinary practices. Instead, it measures the extent to which the industry currently contributes to the size and structure of local economies. This is consistent with practices to analyze mature, existing sectors with broad geographic dispersion in regional economics (16).

Following Miller et al. (1), who analyze Michigan's beer value chain using IMPLAN to estimate direct, indirect, and induced economic contributions, the total economic contributions of the veterinary sector are expressed as the sum of:

$$\begin{array}{l} Total\;Effect=Direct\;Effect+Indirect\;Effect+Induced\;Effect \end{array}$$

Direct effects represent the economic activity directly attributable to the veterinary sector itself. Indirect effects capture inter-industry transactions that arise from veterinary practices' demand for inputs such as pharmaceuticals, laboratory diagnostics, equipment, and business services. Induced effects arise from additional economic activity generated through household spending of labor income earned in both the direct and indirect channels (e.g. groceries, rent, childcare, restaurants, healthcare, and transportation). Because these interdependencies are linear, the total effect can be expressed as a multiplier applied to the direct effect:

$$\begin{array}{l} Total\;Effect\;=\;k\;\times \;Direct\;Effect \end{array}$$

where k ≥ 1 measures the scale of indirect and induced effects. Industries with deeper local supply chains exhibit larger multipliers, while industries that rely more heavily on imports exhibit lower multipliers.

The veterinary services sector is defined using IMPLAN Industry 54194. For each county, IMPLAN provides estimates of employment (full- and part-time jobs) and output (gross receipts or sales). These direct effects represent the economic activity of veterinary practices, including general practice, specialty/emergency care, mobile services, and mixed-animal operations. IMPLAN then applies industry-specific production functions to estimate indirect effects and induced effects. Indirect effects are expenditures on upstream inputs (e.g., wholesale drugs, diagnostic labs, software, professional services, utilities) and reflect the structure of the veterinary supply chain in each county. Induced effects reflect household spending by veterinary employees and workers in upstream industries, generating activity in retail, services, housing, and other sectors. To inform the IMPLAN estimation, starting values on veterinary business counts and baseline industry activity were extracted from DataAxle (17). DataAxle provides establishment-level business listings; we used full-year 2023 records (received in 2025), filtered to NAICS 541940, and aggregated to the county level to construct county establishment counts used in the intensity denominators and to support IMPLAN's initial county-level calibration.

As with all I–O models, the IMPLAN framework relies on several standard assumptions: constant returns to scale, fixed production technology, perfectly elastic supply and fixed price for the year, and linear inter-industry relationships (3). Constant returns to scale implies a doubling of veterinary output requires a proportional doubling of all inputs. Fixed production technology denotes no substitution among inputs, and increases in demand require proportional increases in each specific input. Perfectly elastic supply and fixed prices means industries expand output without supply constraints or price changes. Linear inter-industry relationships assumes that indirect and induced effects are proportional to direct effects. These assumptions are well documented in the I–O literature and should be considered when interpreting results (3).

### County-level implementation

We construct a multi-state dataset covering 331 counties across 10 states using IMPLAN 2023 industry-level detail. The states included are listed in the manuscript's study area description and Table 1; the sample is concentrated in New England (New York, Connecticut, Massachusetts, Vermont, New Hampshire, Maine, Rhode Island and New Jersey), with North Carolina and Indiana added due to additional data availability. For each county, we extract direct and total (direct + indirect + induced) employment and output for IMPLAN Industry 54,194. Total effects are computed using county-specific multipliers, and total economic contributions are reported as the sum of direct, indirect, and induced components. A rural–urban indicator is matched using USDA Rural–Urban Continuum Codes (RUCC) (18), classifying counties with RUCC 1–3 as urban/metropolitan and RUCC 4–9 as rural/nonmetropolitan. This allows for comparison of differences in the scale, intensity, and multiplier structure of veterinary economic activity across rural and urban locations.

**Table 1:** IMPLAN 2023 veterinary services economic contributions by state: direct and total employment and output, overall and by rural/urban county classification.

**State**	***N***	**Employment**		**Output ($ millions)**	
		**Direct**	**Total**	**Direct**	**Total**
**Panel A. Overall**					
Connecticut	8	5,553.00	7,685.45	685.51	1,139.68
Indiana	88	8,049.00	10,146.35	704.73	1,117.86
Maine	16	2,321.00	2,907.77	165.38	277.65
Massachusetts	14	8,294.00	10,795.94	835.90	1,381.53
New Hampshire	10	2,376.00	3,089.36	252.64	398.73
New Jersey	21	9,808.00	13,260.73	1,120.01	1,865.81
New York	61	20,389.00	26,091.33	1,900.61	3,190.61
North Carolina	95	15,842.00	20,234.84	1,241.75	2,106.00
Rhode Island	5	1,710.00	2,229.06	164.50	263.97
Vermont	13	1,096.00	1,471.87	125.44	196.56
**Panel B. Rural counties**					
Connecticut	2	608.00	844.48	76.02	126.39
Indiana	45	1,402.00	1,708.45	114.54	179.00
Maine	11	868.00	1,105.64	66.98	112.45
Massachusetts	3	268.00	332.56	21.57	35.65
New Hampshire	7	752.00	1,010.68	91.61	144.59
New Jersey	0	—	—	—	—
New York	24	1,444.00	1,790.59	115.52	193.93
North Carolina	51	2,014.00	2,566.60	156.21	264.92
Rhode Island	0	—	—	—	—
Vermont	11	654.00	847.12	64.45	100.99
**Panel C. Urban counties**					
Connecticut	6	4,945.00	6,840.97	609.49	1,013.29
Indiana	43	6,647.00	8,437.90	590.19	938.86
Maine	5	1,453.00	1,802.12	98.40	165.20
Massachusetts	11	8,026.00	10,463.38	814.34	1,345.88
New Hampshire	3	1,624.00	2,078.68	161.03	254.14
New Jersey	21	9,808.00	13,260.73	1,120.01	1,865.81
New York	37	18,945.00	24,300.74	1,785.09	2,996.68
North Carolina	44	13,828.00	17,668.25	1,085.54	1,841.08
Rhode Island	5	1,710.00	2,229.06	164.50	263.97
Vermont	2	442.00	624.74	60.99	95.56

Values are from IMPLAN 2023 for the veterinary services industry (IMPLAN Industry 54,194). “Direct” reflects veterinary-sector activity within the county; “Total” reflects the sum of direct + indirect + induced effects. Employment is reported as jobs (full- and part-time). Output is reported in nominal dollars for the IMPLAN 2023 model year. *N* is the number of counties included for each state/panel. Rural–urban county classification is based on USDA rural–urban continuum codes (RUCC), where RUCC 1–3 are classified as urban/metropolitan and RUCC 4–9 are classified as rural/nonmetropolitan.

### Statistical testing

To assess whether there is a difference in the economic contribution between rural and urban veterinary practices, we estimate a log-linear ordinary least squares (OLS) model. The model evaluates whether rural status predicts variation in veterinary economic intensity after adjusting for county-level population and state-specific market conditions. Using OLS, we estimate:


$$\begin{array}{rr} \ln\left(Y_{\text{i}}\right)=\beta_{0}+\beta_{1}Rural\;+\beta_{2}PovertyRate+\beta_{3}UnemploymentRate\\ + & \overset{9}{\underset{s=1}{\sum }}\gamma_{s}Z_{i}+\;\varepsilon_{\text{i}} \end{array}$$

where Y_i_ represents veterinary economic intensity measured as total output or total employment per veterinary establishment per 1,000 residents. Formally, for outcome M ε {total employment, total output}, intensity is constructed as Intensity_i_ = M_i_ / Establishments_i_ / (Population_i_/1,000). This scaling facilitates comparisons across counties of very different size and reduces mechanical confounding between rurality and population; for example, in a county with 50,000 residents, dividing by (Population/1,000) corresponds to scaling by 50. State fixed effects (α_s) control for institutional and macroeconomic differences across states. Rural is an indicator for rural based on the RUCC mapping described above. County poverty rate (share of residents living below the federal poverty threshold) comes from the U.S. Census Bureau's American Community Survey (16), and county unemployment rate comes from the U.S. Bureau of Labor Statistics' Local Area Unemployment Statistics (LAUS) program (19). Standard errors are clustered at the state level, allowing arbitrary correlation within states. Model diagnostics are assessed to ensure proper fit and OLS assumptions are met.

We are interested if there is a rural effect after controlling for the confounding variables of poverty rate and unemployment rate. Many studies find that veterinary demand and income are often the same between rural and urban areas after accounting for population, though this is due to rurality being defined by population. Counties differ in population size and density, so total output or total employment mechanically scale with the number of residents. Without standardization, any estimated “rural effect” would largely reflect scale differences rather than differences in economic intensity of veterinary activity per capita. In other words, without standardizing by population the dependent variable from how large is the economic contribution within the county. After standardizing by population, we can better assess how intense veterinary economic activity is relative to the local population measure. This is important here because using per-capita units reduces the risk that the rural coefficient is simply capturing the fact that rural counties are smaller in population. Standardization helps isolate whether veterinary services generate a residual premium/discount in local economic contribution beyond what would be expected given population size and other county conditions. Given results are per practice per 1,000 residents, the coefficient estimates reflect how “intense” veterinary economic activity is relative to a common local population size. All statistical/econometric computations are performed in R version 4.4.0 (20).

### Data availability

IMPLAN and DataAxle are proprietary data products and cannot be redistributed by the authors. To support reproducibility, we have provide (i) the list of counties/states included in the analysis, (ii) the RUCC-based rural–urban classification and all variable definitions, (iii) the regression equation used to construct intensity measures and estimate models, and (iv) aggregated county-level outputs sufficient to reproduce the reported tables and figures without distributing vendor microdata.

## Results

### State-level economic contributions and rural–urban composition

Across the 10-state, 331-county sample, veterinary services generated 75,438 direct jobs and 97,912.70 total jobs (direct + indirect + induced) (Table 1, Panel A). In output terms, practices generated $7,196.47 million ($7.20B) in direct output and $11,938.40 million ($11.94B) in total output (Table 1, Panel A).

The scale of veterinary economic activity varied substantially across states. New York exhibited the largest totals (20,389 direct jobs; 26,091.33 total jobs; $1,900.61M direct output; $3,190.61M total output), followed by North Carolina (15,842 direct jobs; 20,234.84 total jobs; $1,241.75M direct; $2,106.00M total) and New Jersey (9,808 direct jobs; 13,260.73 total jobs; $1,120.01M direct; $1,865.81M total) (Table 1, Panel A). Smaller states (e.g., Vermont, Rhode Island) exhibited correspondingly smaller totals but still substantial multiplier-driven contributions relative to direct activity (Table 1, Panels A–C; Table 2).

**Table 2:** IMPLAN 2023 state-level multipliers for veterinary services: employment and output (Total ÷ Direct).

**State**	**Employment multiplier**	**Output multiplier**
Connecticut	1.38	1.66
Indiana	1.26	1.59
Maine	1.25	1.68
Massachusetts	1.30	1.65
New hampshire	1.30	1.58
New Jersey	1.35	1.67
New York	1.28	1.68
North Carolina	1.28	1.70
Rhode Island	1.30	1.60
Vermont	1.34	1.57

Multipliers are computed as Total effects divided by direct effects using IMPLAN 2023 county-level results aggregated to the state level for veterinary services (industry 54194). Total effects include direct + indirect + induced.

Rural counties accounted for a minority share of total veterinary economic activity in this multi-state sample. Summed across states with rural counties, rural counties contributed 8,010 direct jobs and $706.90M in direct output, compared with 67,428 direct jobs and $6,489.58M in direct output among urban counties (Table 1, Panels B–C). New Jersey and Rhode Island had no counties classified as rural under the RUCC-based definition used in this dataset (Table 1, Panel B), so their activity appears entirely in the urban panel (Table 1, Panel C).

The rural share of direct activity differed sharply across states (Table 1). Rural counties represented a very small portion of statewide direct employment/output in Massachusetts (3 rural counties; 268 direct jobs; $21.57M direct output) but represented the majority of direct employment in Vermont (11 rural counties; 654 direct jobs vs. 442 in urban counties) and a large share in Maine and New Hampshire (Table 1, Panels B–C). These patterns indicate that rural veterinary activity is concentrated in particular states and does not scale proportionally with the overall size of each state's veterinary sector. In other words, each state is unique and those not analyzed here (*n* = 40) should be investigated.

### State multipliers

State-level multipliers were consistently above one, indicating meaningful indirect and induced spillovers in every state (Table 2). Employment multipliers ranged from 1.25 (Maine) to 1.38 (Connecticut), while output multipliers ranged from 1.57 (Vermont) to 1.70 (North Carolina). In practical terms, these results imply that each direct veterinary job supports roughly 0.25–0.38 additional jobs elsewhere in the local economy, and each dollar of direct veterinary output supports roughly $0.57–$0.70 in additional output through supply-chain and household-spending channels.

### Regression-based rural–urban differences in economic intensity

Regression models estimated on the county-level sample (*N* = 327 counties after removing those missing covariate data) assessed whether rural status predicts veterinary economic intensity after adjusting for poverty, unemployment, and state fixed effects, with state-clustered standard errors. Results are presented in Table 3. Because the dependent variable is log-transformed, effects are reported as percent differences. In the employment-intensity model, rural counties exhibited 12.15% higher modeled intensity relative to urban counties after controls (*p* < 0.001). In the output-intensity model, rural counties exhibited 295.50% higher modeled output intensity on the standardized scale after controls (*p* < 0.001). Unemployment rate was negatively associated with output intensity (−34.98%, *p* < 0.001), while poverty rate showed a positive association (3.79%, *p* < 0.05). In the employment-intensity model, poverty rate was associated with a small increase (0.20%), while unemployment rate was associated with a modest decrease (−1.52%). Together, these results suggest that, after accounting for state differences and local labor-market conditions, rural counties in this sample show higher veterinary economic intensity when measured on a common population scale (per practice per 1,000 residents). Urban counties still have larger total output and employment because they are bigger and have more practices.

**Table 3:** Log-linear regression results from implan output on employment and overall economic output per business per 1,000 people in a county.

**Coefficients**	**Employment**		**Output**	
	**Coefficients (SE)**	**% Difference**	**Coefficients (SE)**	**% Difference**
Intercept	0.059^*^	–	9.394^***^	–
Rural indicator	0.115^***^	12.15%	1.375^***^	295.50%
Poverty percentage	0.002	0.20%	0.037^*^	3.79%
Unemployment rate	−0.015+	−1.52%	−0.430^***^	−34.98%
N	327	–	327	–
State fixed effects	Yes	–	Yes	–
*R* ^2^	0.365	–	0.434	–
F-test	15.048	–	20.043	–
RMSE	0.16	–	1.3	–

Log-linear OLS regressions estimated at the county level with state fixed effects included in all models (not shown). Dependent variables are the natural log of (i) total employment intensity and (ii) total output intensity, where intensity is defined as total (direct + indirect + induced) IMPLAN employment/output divided by the number of veterinary establishments and by (population/1,000). Veterinary establishment counts (“per business”) are from DataAxle full-year 2023 records (received in 2025), filtered to NAICS 541940 and aggregated to the county level. Rural is an indicator for RUCC 4–9 (nonmetropolitan) with RUCC 1–3 as the urban/metropolitan reference group. Poverty rate is from the American Community Survey and unemployment rate is from BLS LAUS. “% Difference” is computed as 100·(exp(β) – 1). Standard errors are clustered at the state level. Significance: + *p* < 0.1, ^*^p < 0.05, ^***^p < 0.001.

Because the dependent variable is the natural log of total intensity, the reported percentage differences are multiplicative: a “295% higher” estimate means the expected total intensity increases about 2.95 times in rural counties than in urban counties, holding controls constant. Again, intensity is defined as total (direct + indirect + induced) employment/output per veterinary establishment per 1,000 residents, so these effects reflect differences in standardized activity rather than differences in county size. In other words, the rural coefficient indicates higher per-practice, per-capita economic contribution—not simply that urban counties are larger. Economically, this suggests that veterinary services function more as a local “anchor” industry in rural counties, with each practice supporting more local employment and spending per resident through both direct activity and downstream multiplier linkages.

## Discussion

This study provides new evidence on how veterinary practices contribute to local economies across rural and urban counties, using a consistent county-level input–output framework applied across 331 counties in 10 states. The results confirm that veterinary services generate substantial economic activity beyond the practice, with indirect and induced spillovers that meaningfully expand employment and output relative to direct effects (21). At the same time, the rural–urban distribution of that activity differs sharply across states. The intensity of veterinary economic activity, once standardized for population and business counts, appears systematically higher in rural counties after accounting for state context and local economic conditions.

A central finding is the large aggregate scale of the sector in the sampled states: 75,438 direct jobs and 97,912 total jobs, alongside $7.20B in direct output and $11.94B in total output. These totals reinforce that veterinary services are not only a healthcare-related industry but also a meaningful contributor to regional labor markets and commercial activity (22). State-level patterns are consistent with expected population and market size effects: New York, North Carolina, and New Jersey account for the largest totals, while smaller states exhibit smaller totals but still substantial total effects relative to direct effects. This aligns with the conceptual basis of contribution analysis: mature industries embedded across regions can generate large aggregate contributions, even when the counterfactual “absence of the industry” is not the question of interest.

The rural–urban decomposition highlights that rural counties account for a smaller share of total sector activity in the multi-state sample, but with pronounced heterogeneity by state. Some states (e.g., Massachusetts) have relatively little rural activity in this dataset, whereas others (e.g., Vermont, Maine, and New Hampshire) show a much larger rural share. Importantly, New Jersey and Rhode Island have no counties classified as rural under the RUCC-based definition used here, emphasizing that rurality is a classification outcome and does not map uniformly across states. These patterns suggest that state-wide discussions of veterinary workforce needs and economic importance can mask where the sector is actually concentrated—often in specific rural counties rather than evenly across the state (5). From a policy standpoint, this reinforces the value of place-based strategies (23): where rural counties account for a large share of veterinary employment and output, local economic exposure to workforce shortages is likely more pronounced.

The multiplier results further contextualize veterinary practices as locally embedded economic contributors. Employment multipliers between 1.25 and 1.38 and output multipliers between 1.57 and 1.70 indicate meaningful supply-chain and household-spending spillovers (21, 24). In practical terms, a direct veterinary job supports an additional 0.25–0.38 jobs elsewhere in the state economy, and each dollar of direct output supports roughly $0.57–$0.70 in additional output through indirect and induced channels. While multipliers are sometimes treated as fixed “sector characteristics,” the observed variation across states is consistent with differences in supply-chain depth, the extent of local purchasing vs. imports (leakage), and regional industrial composition (3). These results support the broader argument motivating rural workforce programs: when a community loses veterinary capacity may not just impact clinical services but also interconnected economic activity that supports other local industries.

Regression results indicate differences in standardized veterinary economic contribution intensity measures between rural and urban counties after controlling for county poverty, unemployment, and state fixed effects. This finding suggests that, conditional on observed covariates, rural veterinary practices may play a disproportionately large role in local economic structure relative to county population. One plausible interpretation is that rural markets may have fewer veterinary providers and thinner service networks, so each practice represents a larger share of local service capacity (5). Another is that, at the practice level, rural practices may operate as broader “generalist” providers, offering a wider range of services with a larger catchment area, concentrating economic activity within fewer establishments. The negative association between unemployment and output intensity is consistent with macroeconomic constraints on household spending and local demand for discretionary or elective veterinary care, which may dampen output per business in weaker labor markets (25). Together, the results point to a distinction that is often lost in public discussion: urban counties account for the majority of veterinary activity in terms of size/scale. However, rural counties can rely more heavily on veterinary practices in terms of intensity on a per-business, per-resident basis. This difference supports the case for targeted rural workforce investments as these efforts should see a higher return per dollar spent.

These findings have direct implications for programs intended to address rural veterinary shortages, such as loan repayment initiatives and other retention incentives (15). If rural counties have higher standardized economic intensity, then the marginal economic consequences of reduced veterinary capacity may be larger than implied by number of veterinarians/businesses alone. Economic development stakeholders (county governments, regional planning bodies, and chambers of commerce) may therefore view veterinary workforce policy not only as an animal-health issue but as part of a broader rural economic resilience agenda (26). More generally, results support the case for integrating veterinary services into regional development planning, particularly in states where rural counties account for a large share of veterinary activity.

Several limitations should be considered when interpreting these results. First, the IMPLAN framework is an accounting-based input–output model that relies on standard assumptions (fixed technical coefficients, constant returns, no price adjustments, and linear inter-industry relationships). These assumptions are well suited for contribution accounting but limit inference about how relationships might change under supply constraints, price changes, or behavioral substitution. Second, multipliers and indirect/induced effects are model-based estimates derived from IMPLAN's regionalization procedures (e.g., RPCs and trade-flow adjustments) and therefore inherit uncertainty and potential measurement error that are not always reflected in conventional statistical tests. Third, the analysis covers 10 states rather than the full U.S., so external validity will depend on how representative these states are of broader rural–urban structures and veterinary market conditions. Fourth, the rural–urban classification uses RUCC codes, which are widely used but necessarily coarse and may mask important within-county variation (e.g., non-rural, non-metro counties) and cross-state differences in how “urban” counties function economically. Finally, while the regressions adjust for poverty and unemployment and include state fixed effects, the specification may still omit relevant confounders, and the results should be interpreted as associations rather than causal effects of rural status.

In addition, these estimates should be read as strong evidence for the study states rather than a claim that the same magnitudes hold nationally. The broader takeaway is the framework and the direction of the rural–urban contrast: when veterinary services are locally embedded, the contribution (and the economic exposure to workforce shortfalls) can be meaningfully different across places. Extending the analysis to additional regions is the next step for testing how general the pattern is.

## Conclusions

The results indicate that veterinary services generate substantial direct and spillover economic activity across states, with meaningful multipliers in employment and output. Urban counties account for most activity in absolute terms, but rural counties appear more economically reliant on veterinary practices on a standardized basis after controlling for local economic conditions and state context. These findings strengthen the evidence base for rural veterinary workforce initiatives by linking workforce capacity to broader local economic contributions and resilience.

Future work can extend this contribution framework in several ways. Expanding to a nationally comprehensive dataset would allow stronger generalization and enable regional heterogeneity analyses (e.g., Appalachia, New England, Upper Midwest). Incorporating measures of clinic structure and service mix—particularly food animal vs. companion animal orientation—could help explain why rural intensity differs and how workforce shortages translate into economic vulnerability. Finally, linking IO-based contribution estimates with practice-level operational data (e.g., staffing, revenues, appointment volumes) could bridge the gap between macroeconomic contribution metrics and the micro-level constraints faced by rural practices, improving the design and targeting of workforce and economic development interventions.

## Data Availability

The data analyzed in this study is subject to the following licenses/restrictions: Both IMPLAN and DataAxle are data sources that must be purchased. Researchers are unable to share data. Requests to access these datasets should be directed to https://implan.com/; https://www.data-axle.com/.
